# Supplementary material for: Strongly Heterogeneous Transmission of COVID-19 in Mainland China: Local and Regional Variation
Source: Front Med (Lausanne). 2020 Jun 19;7:329. doi: 10.3389/fmed.2020.00329 (PMC7317008; doi:10.3389/fmed.2020.00329)
Supplement: Supplementary file 1 [file Data_Sheet_2.pdf]

# Supplementary material

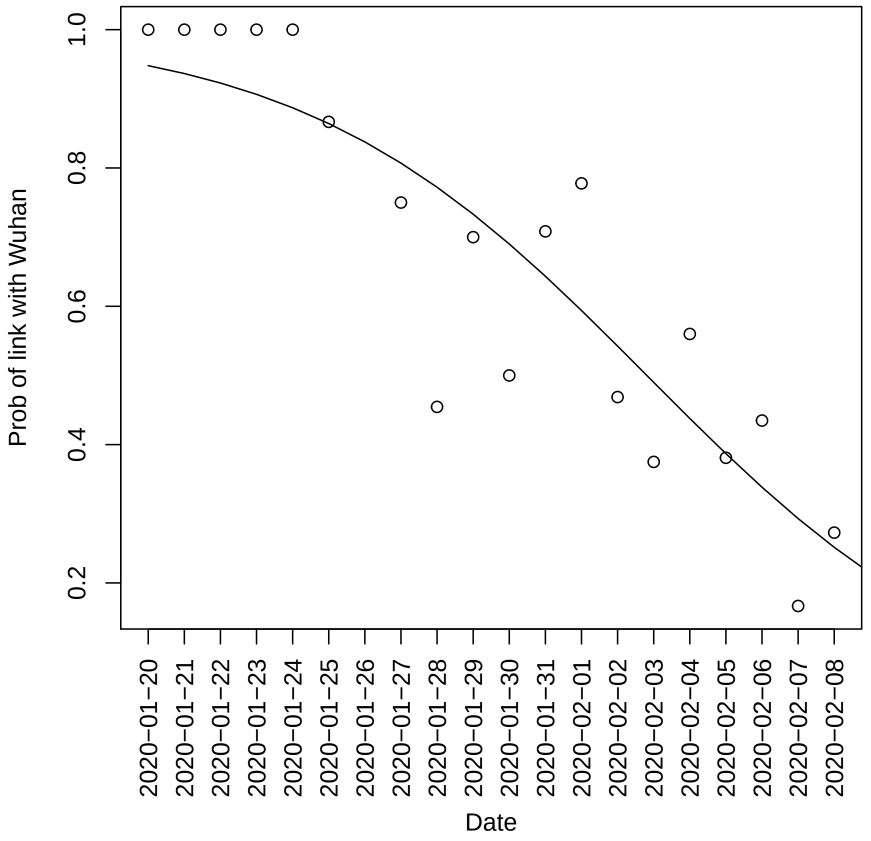


Fig. A1: Model the probability of cases (outside of Wuhan) linking with Wuhan as a logistic function of the date of confirmation. The estimated $\hat{\beta}_0$ is 3.11 (standard error is 0.46) and $\hat{\beta}_1$ is -0.21 (standard error is 0.03).

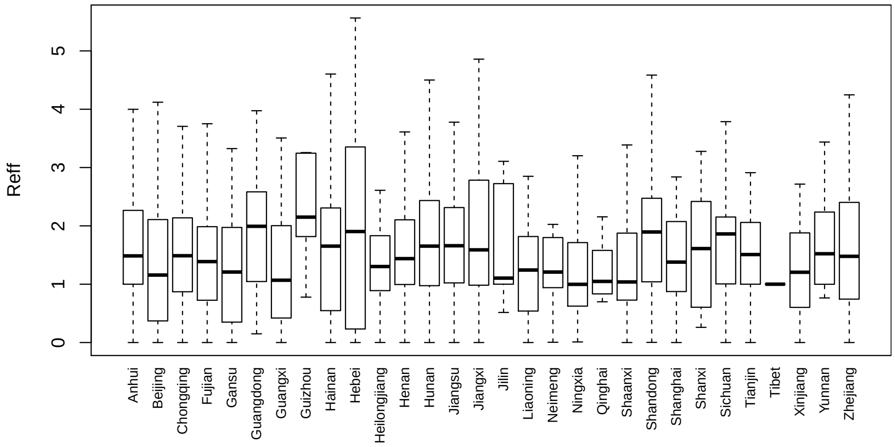


Fig. A2: Boxplot of the reproduction number estimates for the first five days of outbreak in the provinces of China.

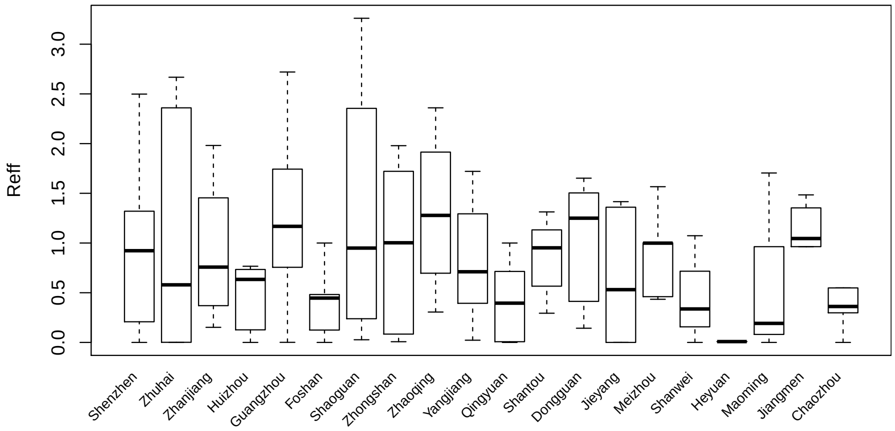


Fig. A3: Boxplot of the reproduction number estimates for the first five days of outbreak in the cities of Guangdong province.

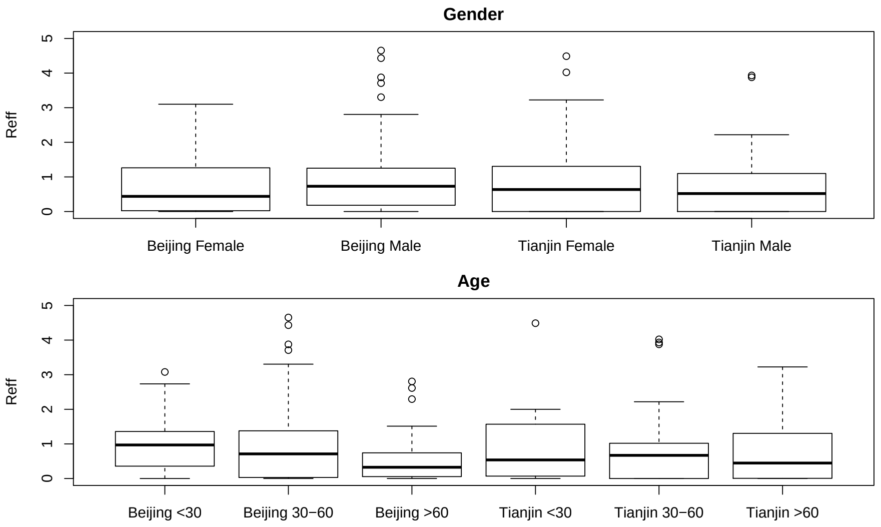


Fig. A4: Estimated reproduction numbers by gender and by age category for Beijing (228 cases) and Tianjin (112 cases).

## Negative serial intervals

The serial interval is variable, and can be as short as one day. Thus, when in an ancestor the time from exposure to onset of shedding is short while they time to symptom onset is long, a descendant may be infected early during the incubation period of the descendant (see additional Fig A5). Then, when the incubation period of the descendant is short, the resulting serial interval (for symptom onset) may be negative. To study the influence of negative serial intervals a translated gamma distribution was used

$$f(\tau) = \text{Gamma}(\tau + \delta|\boldsymbol{\theta}) \tag{A.1}$$

so that the serial interval $\tau$ has a nonzero density on $(-\delta, \infty)$. Although this translated serial interval distribution can be used, the resulting $\boldsymbol{V}$ is not triangular. Loops in the transmission network are not prevented, but as shown in Teunis et al. (14), the resulting sum totals representing $R_{\text{eff}}$ remain unaffected. When the shift $\delta$ is small (1 - 3 days) the resulting reproduction number estimates are not different from those obtained with the unshifted serial interval distribution.

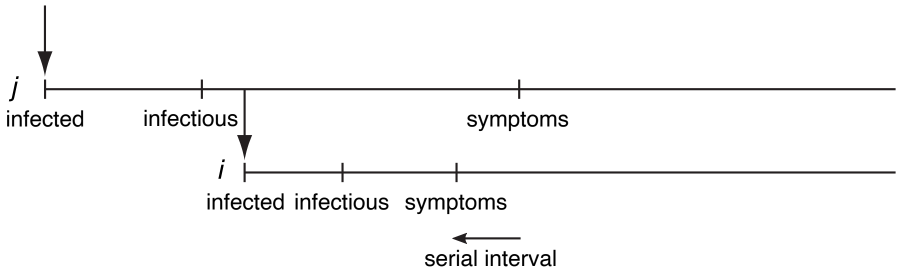


Fig. A5: The serial interval between linked cases. when a subject is infected, there is a latency until they start shedding virus and become infectious to others, then they (may) become symptomatic and can be detected clinically. When subject $j$ infects subject $i$ the time between symptom onset in case $j$ and symptom onset in their descendant case $i$ is the serial interval for onset of clinical symptoms.
